# Convergent genomic signatures associated with vertebrate viviparity

**DOI:** 10.1186/s12915-024-01837-w

**Published:** 2024-02-08

**Authors:** Rhiannon V. Eastment, Bob B. M. Wong, Matthew D. McGee

**Affiliations:** School of Biological Sciences, Monash University, Melbourne, 3800 Australia

**Keywords:** Convergent evolution, Comparative genomics, Viviparity

## Abstract

**Background:**

Viviparity—live birth—is a complex and innovative mode of reproduction that has evolved repeatedly across the vertebrate Tree of Life. Viviparous species exhibit remarkable levels of reproductive diversity, both in the amount of care provided by the parent during gestation, and the ways in which that care is delivered. The genetic basis of viviparity has garnered increasing interest over recent years; however, such studies are often undertaken on small evolutionary timelines, and thus are not able to address changes occurring on a broader scale. Using whole genome data, we investigated the molecular basis of this innovation across the diversity of vertebrates to answer a long held question in evolutionary biology: is the evolution of convergent traits driven by convergent genomic changes?

**Results:**

We reveal convergent changes in protein family sizes, protein-coding regions, introns, and untranslated regions (UTRs) in a number of distantly related viviparous lineages. Specifically, we identify 15 protein families showing evidence of contraction or expansion associated with viviparity. We additionally identify elevated substitution rates in both coding and noncoding sequences in several viviparous lineages. However, we did not find any convergent changes—be it at the nucleotide or protein level—common to all viviparous lineages.

**Conclusions:**

Our results highlight the value of macroevolutionary comparative genomics in determining the genomic basis of complex evolutionary transitions. While we identify a number of convergent genomic changes that may be associated with the evolution of viviparity in vertebrates, there does not appear to be a convergent molecular signature shared by all viviparous vertebrates. Ultimately, our findings indicate that a complex trait such as viviparity likely evolves with changes occurring in multiple different pathways.

**Supplementary Information:**

The online version contains supplementary material available at 10.1186/s12915-024-01837-w.

## Background

Convergent evolution—the process by which similar traits evolve independently—plays a pivotal role in shaping biodiversity [1, 2]. Convergent traits have long been a cornerstone of evolutionary theory because of their ability to inform our understanding of biological complexity, species diversity, adaptation, and selection [3]. The genomic basis of convergent evolution has been investigated in traits as diverse as sensory organs in electric fishes [4], echolocation in mammals [5], and coloration in lizards [6]. While some studies have found that the independent evolution of similar traits may be due to similar genetic changes [4, 5], others show that these independent origins may be due to independent changes in unique regions of the genome [7, 8]. Thus, whether the repeated evolution of complex traits is driven by the same genetic mechanisms remains largely unclear.

One particularly noteworthy example of convergence is viviparity, the process in which offspring are retained within the body of the parent before being born live [9]. Viviparity is an incredibly widespread pattern of reproduction and has evolved from oviparity—the reproductive pattern in which parents lay eggs—more than 150 times in vertebrates. While mammals are perhaps the most well-known group of vertebrates to give birth to live young, viviparity is particularly prominent in the fishes and squamate reptiles, having evolved independently nine times among sharks, 13 times among the bony fishes, and over a hundred times among the squamates [9]. Viviparity is an immensely complex trait involving a number of behavioral, physiological, and molecular changes which must take place to ensure the survival of the embryo within the body of the parent for the entirety of development. These changes, which are often referred to as the parental “adaptations to pregnancy,” include internal fertilization, remodeling of the reproductive tract, and immunotolerance [10, 11]. Each of these processes is critical to offspring growth and survival and is regulated by a complex network of genetic factors.

The increasing interest in the genetic basis of viviparity has revealed a suite of candidate genes and pathways that may be attributed to its origins. Expression of a number of mammalian placental genes have been found to occur during gestation in poeciliid fishes [12], while overlapping gene expression profiles have also been identified between gestating seahorses and the uterus of female mammals and squamates [13]. Much of this overlap occurs in pathways involved in tissue remodeling, nutrient transport, and waste removal, all of which constitute important aspects of vertebrate viviparity. Indeed, an analysis in squamates revealed a number of genes enriched for tissue remodeling to be important for viviparity and embryo retention [14]. Genes involved in angiogenesis and increased oxygen uptake–two vitally important processes to vertebrate viviparity–are also suggested to play an important role in squamate viviparity [15]. Similarly, a number of genes involved in immunotolerance, metabolic processes, and cell-cell signaling have evolved endometrial expression in pregnant mammals [16]. Together, these studies highlight a plethora of genes and their associated pathways that play important roles in the many aspects of vertebrate viviparity, and thus reveal genomic regions that may be targeted in the evolution of viviparity. However, these studies have taken place on small evolutionary timelines and rarely make large-scale phylogenomic comparisons. Thus, the genomic basis of viviparity across the diversity of vertebrates remains to be resolved.

Few studies have investigated the molecular drivers of viviparity at a genome-wide level across a breadth of vertebrates. Transcriptomic data from 8 viviparous vertebrates across the squamates, mammals, and sharks found no overlap in gene expression, suggesting that viviparity may have evolved via independent genetic changes [17]. Consistent with this, an analysis in Cyprinodontiforme fishes found no excess of molecular convergence relating to viviparity, again indicative of unique molecular mechanisms driving these transitions [18]. Together, these studies provide important insights on the molecular drivers of viviparity in the context of convergent evolution and suggest that the independent origins of viviparity may be driven by unique molecular mechanisms. However, given the limited taxonomic sampling, such studies are unable to address changes that occur on a broader scale. As such, it is still unclear if there is a common molecular signature associated with the transition to viviparity among vertebrates.

Here, we used whole genome data to investigate the molecular basis of viviparity across an evolutionary timeline spanning more than 400 million years [19]. We sequenced and assembled seven new ray-finned fish genomes and used these in combination with 45 existing vertebrate genomes across the ray-finned fishes, lobe-finned fishes, sharks, reptiles, and mammals to account for a total of 17 independent transitions to viviparity (see Additional file 1: Supplementary Information and Additional file 2: Table S1) [9, 19–114]. This whole-genome data was then used to make phylogenetic, genomic, and proteomic comparisons between viviparous and oviparous species. In doing so, we reveal a number of convergent molecular changes in several distantly related viviparous lineages, but do not find a molecular signature common to all viviparous vertebrates. Ultimately, this suggests that the transition to viviparity is likely driven by unique genomic changes.

## Results and discussion

**Fig. 1:**
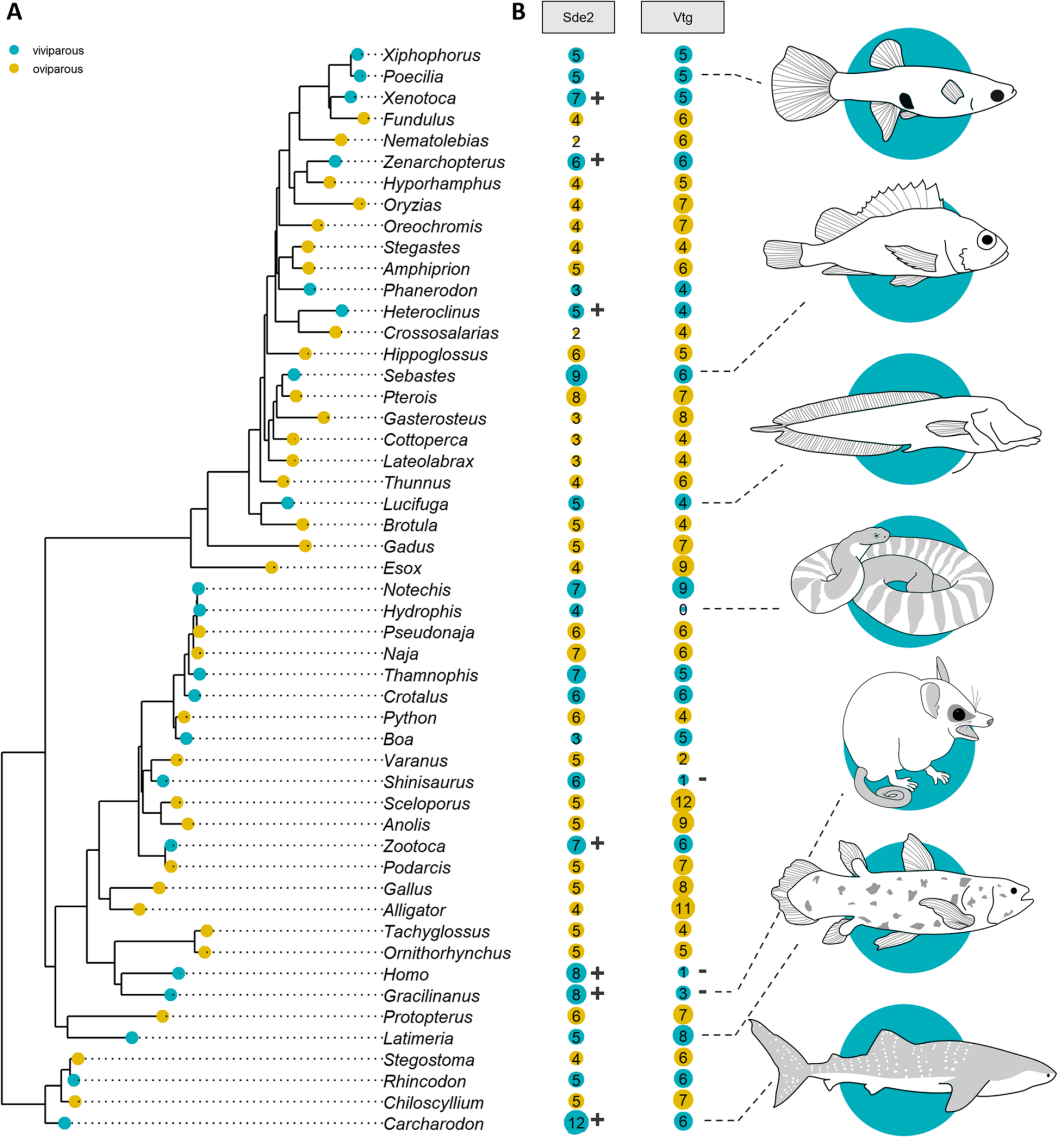
The evolution of protein family size and reproductive mode in vertebrates. **A** Displayed here is a species tree topology that reflects the neutral phylogenetic model generated using fourfold degenerate sizes from 51 vertebrate species, with species names displayed as genera. **B** The number of sequences associated with the protein families ubiquitin fold N-terminal domain of silencing defective 2 (Ubi-N-Sde2) and the beta-sheet shell domain of vitellogenin (b-sheet-shell-Vtg) among viviparous and oviparous vertebrates

Our whole-genome analysis of viviparity reveals a signature of convergence associated with large-scale alterations to the protein repertoire. Each of the 51 vertebrate genomes was aligned to the Pfam database: an online repository containing the annotations and multiple sequence alignments of over 19,000 protein families [115]. In doing so, we determined the size of 7467 protein families for each of the vertebrates in our dataset. We define the size of a protein family as the number of non-overlapping sequences showing strong evidence of homology to those in the Pfam database. We then assessed differences in the size of each protein family between viviparous and oviparous vertebrates using both phylogenetically corrected generalized linear mixed modeling and Bayesian regression modeling (see Additional file 1: Supplementary Information and Additional file 3: Fig. S1) [19–34]. Neutral and dated phylogenetic models were implemented to account for relationships among taxa (Fig. 1A and Additional file 4: Fig. S2). In doing so, we identified 15 protein families with evidence of expansion or contraction among viviparous lineages (see Additional file 5: Table S2). The correlation between sequence number and reproductive mode in these 15 families suggests that large-scale genomic changes, specifically to the sizes of protein families, may play an important role in facilitating the transition to viviparity. Expansion of several gene families related to lipid and energy metabolism were found to be associated with viviparity in insects, which may play a role in the transfer of nutrients from parent to offspring during gestation [116]. Gene family expansion has also been linked to viviparity in the Korean rockfish (*Sebastes schlegelii*), in which expansion of the bradykinin B2 receptor (Bdkrb2) family is suggested to contribute to the adaptation to viviparity through its role in fertilization and hatching [117]. Our results add to a mounting body of evidence which shows that the contraction and expansion of protein families can act as a strong force in the evolution of novel traits, such as viviparity [16, 118–120].

We find an example of convergence within the ubiquitin fold N-terminal domain of silencing defective 2 (Ubi-N-Sde2) family (Fig. 1B), which was expanded in six viviparous lineages relative to closely related oviparous taxa. Expansion was observed in ray-finned fishes (*Heteroclinus perspicillatus*, *Xenotoxa eiseni* and *Zenarchopterus caudovittatus*), sharks (*Carcharodon carcharis*), squamate reptiles (*Zootoca vivipara*), and mammals (*Gracilinanus agilis* and *Homo sapiens*). Sde2 is a ubiquitin-like protein that plays a critical role in DNA replication and transcription [121] and is highly expressed in adult reproductive tissues, oocytes, and eggs [122, 123]. The ubiquitin-fold of Sde2 is particularly important in biological regulation, as its cleavage from Sde2 allows for the remaining protein to attach to the spliceosome. This then facilitates a plethora of fundamental cellular processes such as chromatin silencing and gene expression and is integral to the maintenance of genomic stability [124, 125].

We traced the evolution of Ubi-N-Sde2 across 51 vertebrates and found that expansion likely arose repeatedly and independently between taxa (see Additional file 6: Fig. S3). We further investigated the nature of protein family expansion in mammals by tracing the location of Ubi-N-Sde2 sequences in the human (*H. sapiens*), opossum (*G. agilis*), and platypus (*Ornithorhynchus anatinus*) genomes. Doing so revealed the presence of these motifs in Sde2, as anticipated, but also in ubiquitin and ubiquitin-like genes (see Additional file 7: Fig. S4). In both oviparous and viviparous mammals, Ubi-N-Sde2 motifs were found in the highly conserved gene ubiquitin-C (*UBC*), which plays a critical role in a multitude of biological processes, such as cell signaling, DNA repair, and gene expression [126]. Interestingly, we identified additional Ubi-N-Sde2 motifs in viviparous mammals which were located in novel ubiquitin-like regions of the genome.

In the case of humans, expansion of Ubi-N-Sde2 may involve pseudogenes, with 3 of the 8 human motifs found in ubiquitin A-52 residue ribosomal protein fusion product 1 pseudogene 1 (*UBA52P1*), ubiquitin A-52 residue ribosomal protein fusion product 1 pseudogene 5 (*UBA52P5*), and ubiquitin B pseudogene 4 (*UBBP4*). Orthology tests suggest that none of these pseudogenes are present in the marsupial and monotreme mammals. We investigated the evolutionary forces driving the expansion of Ubi-N-Sde2 sequences in pseudogenes by computing the rate of nucleotide substitution for sequences and found no evidence to suggest that Ubi-N-Sde2 sequences, be they protein coding or pseudogenic, were evolving at different rates; pseudogenic Ubi-N-Sde2 sequences are evolving at the same rate as their protein coding counterparts. Traditionally, pseudogenes were thought to be subject to a high number of mutations which often render them non-functional [127, 128]. Here, we find no evidence to suggest that pseudogenic Ubi-N-Sde2 sequences experience elevated substitution rates, which may allow them to resist degradation and thus hints at their biological importance [128]. These results substantiate the emerging body of literature which highlights the role of pseudogenes in many biological functions, such as gene regulation [129, 130].

We also found evidence for contraction of some protein families within viviparous lineages. The protein family comprising the beta-sheet shell domain of vitellogenins (b-sheet-shell-Vtg) is noticeably contracted in two viviparous lineages (Fig. 1B), including in placental and marsupial mammals (*H. sapiens*, *G. agilis*), as well as the Chinese crocodile lizard (*Shinisaurus crocodilurus*). Vitellogenins are the primary precursors of egg yolk proteins and play a major role in fetal nourishment in oviparous species [131]. However, egg yolk also remains a vital source of fetal nutrition in many viviparous vertebrates, such as sharks [132, 133]. In these species, parents provision their eggs with yolk to support embryos for either part or all of gestation. In other viviparous species, such as mammals, parents incubate their embryos in an absence of yolk and have thus lost the need for vitellogenins [134]. Therefore, the observed contraction in vitellogenins in viviparous mammals and the Chinese crocodile lizard may not necessarily be a requirement of viviparity, but rather an association with their specific mode of nutrient provisioning during pregnancy. Traditionally, gene loss was considered a weak evolutionary force that had little to no impact on phenotypic diversity [135]. Here, we substantiate recent findings which suggest that the loss of a trait may be followed by loss of related genes [134] and show that gene loss may play a fundamental role in shaping life on Earth through inflicting genetic change [136].

We used PhyloAcc to investigate changes in the evolutionary rate of conserved protein-coding regions (CDS, *n*=858) shared between 27 vertebrates with 14 independent origins of viviparity [137, 138]. Here, comparisons were made between a smaller number of taxa to maximize the number of syntenic regions, while still accounting for a high number of evolutionary transitions across a breadth of vertebrates. In doing so, we identified 20 CDS with accelerated substitution rates specifically in viviparous lineages. Gene ontology (GO) analysis revealed no signatures of enrichment across the viviparous-accelerated CDS. However, we did find evidence of convergent acceleration in 2 CDS, in which the substitution rate was elevated in two or more viviparous species for the same element. Among these genes was even-skipped homeobox 1 (*EVX1*)—a key regulator of embryonic development, with a particularly important role in anterior-posterior patterning and implantation [139]. To determine if positive selection may account for elevated substitution rates among viviparous species, we determined the strength of selection on sites among each of the accelerated CDS which revealed no significant differences between oviparous and viviparous species.

We additionally assessed whether viviparous species experience convergent shifts in amino acid substitutions but found no evidence of positive selection. Both maximum likelihood (PAML) and Markov chain Monte Carlo (BAli-Phy) approaches were used to assess differences in substitution rates between viviparous and oviparous species [34, 140]. While mutations in the same amino acid position do occur among some closely related taxa with convergent traits [141], they are exceedingly rare among species that occupy different taxonomic orders [7, 142]. This is likely due to the infrequent role of protein-coding regions in vertebrate morphogenesis; changes to the regulatory networks of genes are suggested to play a far more prominent role [137, 143, 144].

**Fig. 2:**
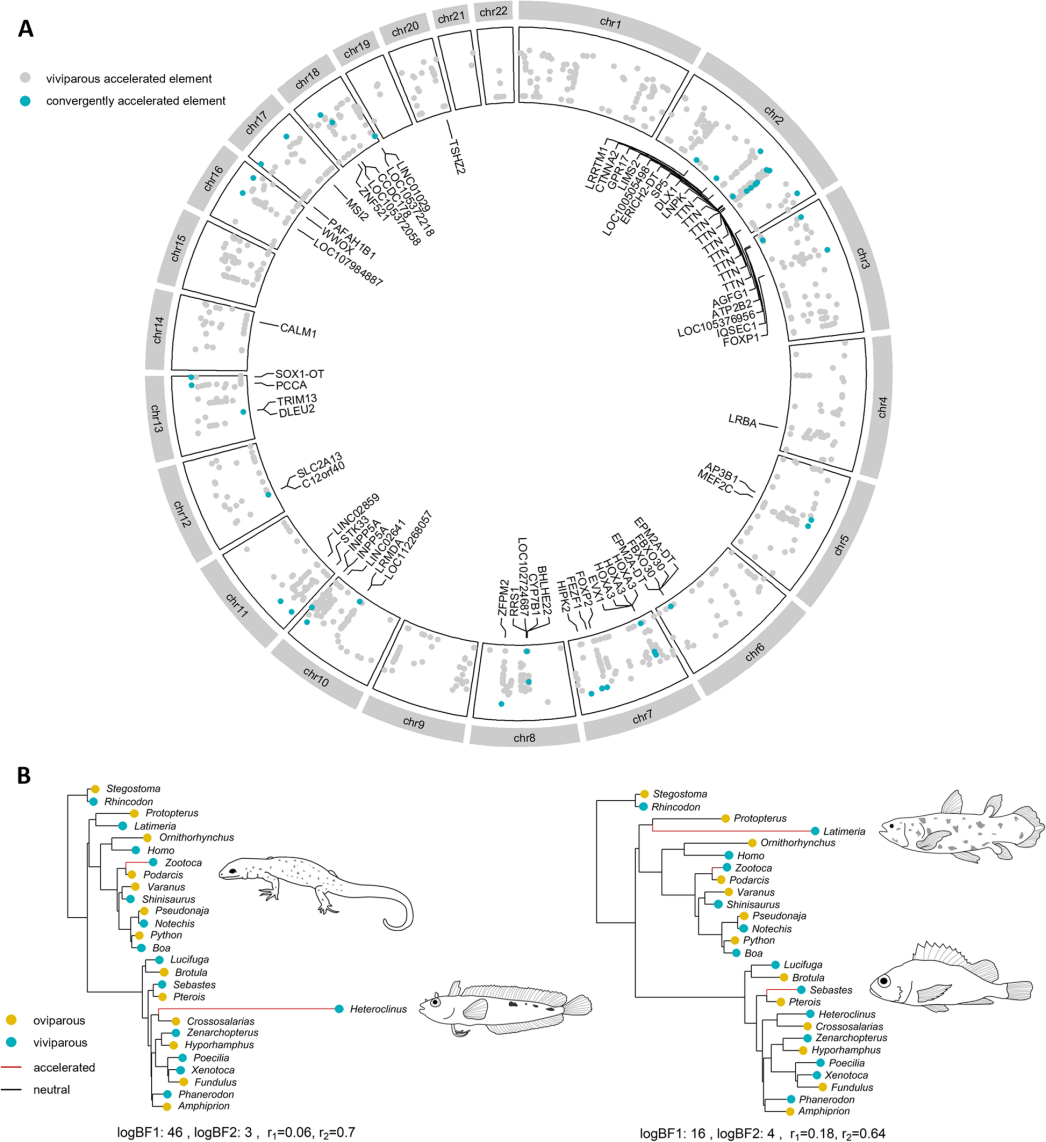
Acceleration of noncoding elements in viviparous species. **A** The location of syntenic introns and UTRs among 27 vertebrates with respect to the coordinates of the *H. sapiens* genome, with their associated genes annotated. **B** Trees for two convergently accelerated elements among viviparous species. The Bayes factors (BF1 and BF2; see Additional file 1: Supplementary Information), conservation rates (r1) and accelerated rates (r2) are indicated for each element

Noncoding regions of genes—such as introns and untranslated regions (UTRs)—are more susceptible to functional changes than protein-coding regions, as they do not have to conform to a strict triplet code of nucleotides [145]. To test whether introns and UTRs play a role in the evolution of viviparity, we generated 1598 intron and UTR sequence alignments (≥50 base pairs) shared between our 27 vertebrate genomes. Changes in the conservation of introns and UTRs were assessed using PhyloAcc [137, 138] to reveal 55 regions with accelerated substitution rates in viviparous lineages (Fig. 2A). GO analysis revealed that viviparous-accelerated introns and UTRs were enriched in genes associated with developmental processes, anatomical structure morphogenesis, and transcription regulator activity, all of which have a functional relevance to viviparity [9].

We find evidence of convergently accelerated substitution rates in introns and UTRs across the full range of vertebrate diversity. Of the 55 viviparous-accelerated introns and UTRs, eight showed evidence of convergent acceleration, in that the region had an elevated substitution rate in two or more viviparous lineages (Fig. 2B). Convergently accelerated elements were present in key developmental genes, including adaptor-related protein complex 3 subunit beta 1 (*AP3B1*), sp5 transcription factor (*SP5*), and zinc finger protein 521 (*ZNF521*). *AP3B1* is involved in organ biogenesis and is thought to be important in sexual development and parental investment in mice [146, 147]. Both *SP5* and *ZNF521* are transcription factors that play critical roles in cell differentiation and are key drivers of morphogenesis and neural cell differentiation, respectively [148, 149]. Ultimately, our findings support the notion that developmental genes are targeted repeatedly in the evolution of complex traits [137], such as viviparity.

Our results demonstrate that molecular convergence can occur across the vertebrate Tree of Life. While we did not find evidence of universal acceleration of substitution rates among viviparous species for any single region, we did find that convergent acceleration occurs repeatedly in subsets of distantly related vertebrates, many with a common ancestor dating back at least 420 million years [19]. We additionally show that regulatory changes to both coding and noncoding regions of the genome may play a key role in the evolution of viviparity. While several studies have investigated the genomic basis of viviparity, they often do so on small evolutionary timelines, with comparisons being made between animals that fall within a single family rather than those that span distant taxonomic scales [16, 150]. These studies provide important insights on the molecular drivers of viviparity within a lineage but are unable to address those that occur on a broader scale. By utilizing whole-genome data across a wide array of taxa, we demonstrate that conserved regions of the genome may play a significant role in the recurring transition to viviparity among vertebrates.

## Conclusions

Despite many striking examples of convergence in distantly related lineages, the molecular basis of convergence is rarely examined across large evolutionary distances, and seldom in a genome-wide fashion [137, 145]. Fortunately, rapid advancements in sequencing technology coupled with reductions in sequencing costs has greatly increased genomic datasets [151], which can provide a unique opportunity to examine the molecular drivers of convergence across the Tree of Life [152]. Here, we utilized a combination of newly sequenced and publicly available genomes to investigate the molecular basis of viviparity and examine whether its independent origins among vertebrates are driven by similar genetic changes. We identify candidate genes and pathways with signatures of convergence in some viviparous lineages, but ultimately conclude that different molecular mechanisms are likely utilized in the transition to viviparity. We show that it is possible to recover signatures of molecular convergence on macroevolutionary timescales, and thus anticipate that the study will encourage others to explore the molecular dynamics of convergent evolution across large portions of biodiversity. Ultimately, our findings suggest that large-scale analyses of convergent evolution are likely to be vital for identifying the genomic basis of complex evolutionary transitions [152–155].

## Methods

### Sequencing and assembly of new teleost genomes

Genomic DNA was extracted from samples belonging to *Crossosalarias macrospilus*, *Heteroclinus perspicillatus*, *Hyporhamphus melanochir*, *Phanerodon vacca*, *Pterois antennata*, *Xenotoca eiseni*, and *Zenarchopterus caudovittatus*. The *P. vacca* sample was sent to a commercial sequencing provider (Phase Genomics, Seattle, WA), which produced a 10X Chromium assembly. All other samples were sent to Deakin Genomics (Geelong, VIC), where libraries were prepared using PCR-free protocols, then sequenced on S4 flowcells using Illumina Novaseq. Following sequencing, insert size distributions were identified using BBMerge from the BBTools package v17.12 [156]. Samples were subsequently assembled using MaSuRCA v4.0.3 [157].

### Whole genome alignments

LAST was used to generate two multiple-genome alignments: a “default” alignment comprising 27 vertebrate genomes, and an “extended” alignment comprising 51 vertebrate genomes (see Additional file 2: Table S1) [20, 37–114]. Both alignments comprised the seven teleost genomes generated above, and additional genomes obtained from NCBI. The “default” dataset accounted for 14 evolutionary transitions and was used to analyze differences in substitution rates as it provided a higher number of syntenic regions than the “extended” dataset. The “extended” dataset was used to analyze differences in protein family sizes as it did not require synteny.

Genome statistics, such as BUSCO scores, genome lengths, and N50 values were calculated using gVolante [158], except in the case of *Protopterus annectens*, in which statistics were obtained from existing data [93].

The reference genome—belonging to *H. sapiens*—was prepared for alignment using “lastdb” from the LAST software package [20]. All other genomes were pre-processed to improve the efficacy of alignment. To do this, repeat sequences were hard masked using BBMask from the BBTools v38.81 suite [159]. Masked genomes were then broken into multiple contigs using the custom perl script “break_scaffolds_by_Ns.pl” [160], which breaks scaffolds at regions containing ≥ 100 unspecified characters (i.e., “Ns”). We additionally removed sequences shorter than 250bp using BBDuk from BBTools v38.81 and removed those representing alternate haplotypes using Redundans [161]. Redundans was run in high identity mode (identity = 0.9) to prevent rearrangements and short-read scaffolding, and to extract contigs spanning ≥ 320bp.

All genomes were individually aligned to the reference using a series of programs from LAST. First, “last- train” was used to identify the rates of insertion, deletion, and substitution between the focal genome and the reference genome. Preliminary alignments were then generated using “lastal,” which were subsequently refined using “last-split.” “last-postmark” was used to remove alignments caused by simple sequences, and “maf-sort” was used to sort alignments by sequence name, strand, and position. Finally, pairwise alignments were joined to form the “default” and “extended” alignments using “maf-join.”

### Extraction and alignment of coding and noncoding regions

Coding and noncoding sequences from each of the multiple-genome alignments were extracted to yield individual alignments composed of either protein-coding sequences (CDS) or introns and UTRs, respectively. To do this, we identified CDS, introns, and UTRs in the *H. sapiens* genome using the associated annotation file from NCBI. We then used GFFUtils v0.12.0 [162] to generate two new annotation files, one which defined the location of CDS, and the other that identified the location of whole genes. CDS were then removed from genes using the “subtract” function from bedtools v2.26.0 [163] to yield the location of introns and UTRs.

We extracted sequence alignments corresponding to CDS, and introns and UTRs using MafFilter v1.3.1 [28]. CDS alignments were exported on the condition that they spanned ≥ 200bp and represented the complete feature. We required that introns and UTRs simply span ≥ 50bp. All coding and noncoding alignments were screened for duplication and/or overlap. Gaps and unspecified characters were removed from each of the CDS and noncoding sequences before realigning each using MAFFT v7.310 [164]. MAFFT was run using the iterative refinement method, with the maximum number of iterations set to 1000.

### Species tree inference

We generated a species tree topology for both the “default” and “extended” datasets using IQ-TREE v2.0 [165]. To do this, we concatenated the realigned introns and UTRs from each of the respective datasets using AMAS [166]. We then ran IQ-TREE with a General Time Reversible (GTR) and FreeRate model [167, 168], using the appropriate concatenated introns and UTRs as input*.* The topology from the “default” dataset was used as a constraint for the “extended” dataset. The resulting tree topologies were rooted at the node connecting Chondrichthyes and Osteichthyes using the “root” function from the R package “ape” [19, 169].

### Neutral phylogenetic model

We created neutral phylogenetic models for both the “default” and “extended” datasets using fourfold degenerate (4d) sites from each of the whole-genome alignments (see Additional file 4: Fig. S2). 4d sites were extracted from the alignments using “msa_view” from the package PHAST v1.4 [170], using the *H. sapiens* genome as a reference. We used the 4d sites, in combination with the appropriate rooted species tree, to generate a neutral model of evolution with phyloFit from the PHAST package. Ancestral branches were labeled using “tree_doctor,” also from the PHAST package.

### Time tree

Two time trees were generated using IQ-TREE v2.0 [165]. Here, the branches of the neutral phylogenetic model were rescaled by dating the ancestral nodes according to Benton et al. [19] in one of two ways (see Additional file 4: Fig. S2). The first time tree was dated according to the soft maximum ages of divergence outlined in Benton et al. [19], while the second time tree was dated according to the minimum ages. Each tree was generated using concatenated alignments of conserved introns and UTRs and the GTR+F+R5 model, which was identified by Model Finder as the model of best fit [171, 172]. Both time trees contained polytomies throughout the spiny-finned fishes, which is expected given the high levels of incomplete lineage sorting due to explosive diversification in the Cretaceous [173, 174].

### Estimating protein family sizes

We analyzed the evolution of protein family sizes across taxa in our “extended” dataset to determine whether the size of protein families is implicated in the transition to viviparity. To do this, we individually aligned all 51 genomes to the Pfam database [115, 175]. Genomes were prepared for alignment using BBMap from the BBTools v38.81 suite [159], in which sequences longer than 50,000bp were broken into multiple sequences of 50,000bp in length, and sequences shorter than 1000bp were removed. The resulting sequences were then aligned to the Pfam database using “lastal” from the LAST package to generate a total of 51 pairwise alignments in blasttab format [20].

Alignments were then filtered to remove all sequence alignments with an E-value ≥ 1e−10, before removing overlapping sequences using the “GenomicRanges” package in R [176]. Each blasttab was first converted to a GRanges object using the “GRanges” function, with each alignment corresponding to a unique genomic region. We then used the “reduce” function to remove regions of overlap for a particular protein family, effectively merging all overlapping alignments into one GRanges entry. The resulting Granges objects were then converted to count tables, each one outlining the number of unique sequences within a particular family for any given species.

### Analysis of protein family sizes

We used the R package “phyr” to investigate the relationship between protein family size and viviparity, while accounting for the phylogenetic relationships among the 51 species in our “extended” dataset [177]. We employed a Phylogenetic Generalized Linear Mixed Model (pglmm) to test this, setting reproductive mode (i.e., viviparity vs oviparity) as the predictor, and species (i.e., phylogeny) as the random factor. We ran the pglmm three times, each time using either the neutral phylogenetic model, the time tree based on minimum ages, or the time tree based on maximum ages. The type of phylogeny (i.e., the neutral phylogenetic model and time trees) used in this analysis did not alter the results, and thus we present the results in which the neutral phylogenetic model was used as the random factor in the pglmm.

We ran the pglmm for all protein families using a Poisson distribution. To refine the dataset and detect families with the strongest evidence of correlation between protein family size and viviparity, we extracted counts for families with *P* ≤ 0.05 and excluded families of mitochondrial and ribosomal origin. Results were only deemed significant upon further analysis using Bayesian methodology, described below.

We produced a phylogenetically corrected Bayesian regression model using the “brms” package in R to test for significant differences in protein family size between viviparous and oviparous species, while accounting for their relative phylogenetic positions [178]. To begin, we generated a variance-covariance matrix from the neutral phylogenetic model using the “vcov” function from the R package “ape” [169]. We then used this matrix as a random factor in the model, which utilized a Poisson distribution. The model was run for 4000 iterations with 4 cores and default priors. Results were considered significant if the 95% credible intervals of the effect size did not overlap zero.

### Mapping the evolution of Ubi-N-Sde2

We further analyzed the expansion of the protein family Ubi-N-Sde2 to determine (1) whether duplication in viviparous species is ancestral (that is, whether expansion occurs prior to the transition to viviparity), (2) the genomic location of Ubi-N-Sde2 sequences, and (3) the evolutionary rate of Ubi-N-Sde2 sequences.

To map the evolution of Ubi-N-Sde2 across our “extended” dataset, we obtained the Ubi-N-Sde2 nucleotide sequences for each species using the “getfasta” function from bedtools v2.26.0 [163], ensuring that strandedness was enforced. Sequences were then clustered and subsequently aligned using MAFFT v7.310 [164]. These alignments were then sequentially aligned to one another, from longest to shortest, using the “add-fragment” function in MAFFT. The resulting alignment was used to generate a phylogenetic tree, which was produced in IQ-TREE using a GTR and FreeRate model [Additional file 6: Fig. S3 and [165, 167, 168].

We further examined the expansion of Ubi-N-Sde2 in mammals to determine the genomic location of sequences, as well as whether they were protein-coding or pseudogenic, using the UCSC Genome Browser [32]. For each gene containing sequence fragments of Ubi-N-Sde2, we identified orthologous genes in the remaining mammals using the UCSC Genome Browser, minimap2 [33], and the “maf- cut” program in LAST [20] (see Additional file 1: Supplementary Information) [20–34]. We additionally investigated the presence of the human pseudogenes *UBBP4*, *UBA52P1*, and *UB52P6* in other placental mammals by mapping them to the genome of the house mouse (*Mus Musculus;* GCF_000001635.27) using minimap2 [33], but found no orthologous sequences. The resulting coordinates from each of the tests of orthology were then used to obtain the nucleotide sequences corresponding to each gene using the “getfasta” function in bedtools, ensuring that strandedness was enforced. To assess the potential causes and consequences of Ubi-N-Sde2 expansion in mammals, we generated whole-gene alignments for *UBC* and *UBB* using BAli-Phy (5000 iterations) and the iterative refinement method in MAFFT (1000 iterations).

Finally, we sought to investigate the rate of evolution of Ubi-N-Sde2 sequences in viviparous mammals and determine whether positive selection may account for the expansion of the Ubi-N-Sde2 family. We generated an amino acid alignment for Ubi-N-Sde2 sequences in mammals using MACSE v2.06, which is a codon-aware aligner that aligns protein-coding regions without altering the underlying codon structure [179]. By accounting for codon structure, MACSE often introduces frameshifts and stop codons to sequences which are generally incompatible with other software programs. To account for this, we used the exportAlignment program in MACSE to replace all frameshifts with gaps, all stop codons at the end of sequences with gaps, and all internal stop codons with “N” [179]. The resulting nucleotide alignments were used to generate phylogenetic trees, which were produced using IQ-TREE with a GTR and FreeRate model [165, 167, 168]. Trees were then rooted at the node connecting the longest branch using the “root” function from the R package “ape” [169].

We investigated the rate of evolution of Ubi-N-Sde2 sequences using the above alignment and the codeml program in PAML v4.9 [34]. We ran three PAML models in total: M0, M1, and M2. We first ran model M0, which fits a single dN/dS to each branch. We then ran the free-ratio branch model (M1), which fits a unique dN/dS to each branch. Finally, we ran model M2, which fits a separate dN/dS to foreground branches (i.e., those leading to expanded viviparous species) and background branches. Each model tested for positive selection in one of two ways. First, we tested for positive selection on every branch leading to viviparous mammals (i.e., all branches leading to *H. sapiens* and *G. agilis*). Second, we tested for positive selection on branches leading to each species separately (that is, testing each branch/sequence individually). All alignments were run with their respective clade-specific phylogenetic tree, above. We then constructed likelihood ratio tests comparing models M0 and M2, and models M1 and M2.

### Analysis of conserved coding and noncoding elements

We assessed the rate of evolution of coding and noncoding regions across our “default” phylogeny using PhyloAcc [137, 138]. Specifically, we aimed to identify elements with altered substitution rates in viviparous species relative to oviparous species. PhyloAcc employs three models: the null model, in which the element of interest is conserved in all species, the accelerated model, in which the element is accelerated in the target species, and the full model, in which the element is accelerated in all species. It uses Bayes Factor criteria to compare the accelerated and null models to identify regions that are accelerated in the target species, regardless of the remaining species (Bayes Factor 1; BF1). It additionally compares the accelerated and full models to identify regions that are accelerated specifically in the target species (Bayes Factor 2; BF2). PhyloAcc takes as input a phylogeny, a transition rate matrix for bases under a neutral model, a multiple alignment file containing the concatenated sequences of interest, and a partition file that details the position of each sequence in the concatenated alignment file. The neutral model generated from the “default” dataset was used for both the phylogeny and transition matrix.

Alignments corresponding to coding and noncoding regions of the *H. sapiens* genome were extracted from the “default” alignment using MafFilter [28]. All gaps and unspecified characters were then removed from alignments, which were subsequently realigned using MAFFT [164]. We then generated alignment files corresponding to aligned coding and noncoding sequences, respectively, by concatenating the individual alignments using AMAS [166]. By default, AMAS creates a partition file describing the new coordinates of each of the concatenated elements. Partition files were manually converted to 0-based BED files for compatibility with PhyloAcc.

We ran PhyloAcc with default parameters, with the target species set to viviparous species. We define viviparous-accelerated elements as those with BF1 ≥ 10, BF2 ≥ 1, and a posterior probability of acceleration ≥ 0.9 for at least one viviparous species. We then define convergently accelerated elements as those with BF1 ≥ 10, BF2 ≥ 1, acceleration in at least 2 viviparous species (i.e., posterior probability ≥ 0.9), and additionally require that no oviparous species shows evidence of acceleration (i.e., posterior probability < 0.9).

We tested whether viviparous-accelerated elements were enriched for gene ontology (GO) terms using GOrilla [180]. To do this, we first generated “target” lists of genes containing the names of genes associated with the accelerated elements. This was achieved using the “intersect” function from Bedtools and the “gtf_extract” function from GFFUtils [162, 163]. Using these same methods, we generated two “background” lists of genes, containing either the names of genes associated with all coding or noncoding regions in our alignment.

#### Analysis of positive selection on protein-coding regions

We tested coding alignments for evidence of positive selection to determine whether (1) positive selection may account for rate variation in viviparous species, and (2) whether viviparous taxa experience convergent shifts in amino acid substitutions. Coding alignments were extracted from the “default” whole-genome alignment as above and were then split into files containing “reliable” and “less-reliable” sequences using “msa_view” from the PHAST package [170]. For each alignment, we deemed the *H. sapiens* sequence as “reliable,” and all other sequences as “less reliable.” We then realigned each coding alignment using the refineAlignment program in MACSE v2.06 [179], generating both a nucleotide and amino acid alignment for each coding region. Alignments were refined using the exportAlignment program in MACSE to replace all frameshifts with gaps, all stop codons at the end of sequences with gaps, and all internal stop codons with “N.” We then tested all refined coding alignments for evidence of positive selection in all viviparous species using the codeml program in PAML v4.9 [34]. All alignments were run with the neutral phylogenetic model generated from the “default” dataset. Given that the use of a single species tree to identify changes in substitution rates can result in incorrect inferences due to the underlying loci having a different topology, we quantified discordance using both gene concordance factors (gCF) and site concordance factors (sCF) in IQ-TREE v2.2.2.7 (Additional file 9: Fig. S5) [165]. To do this, single-locus trees were generated using either coding or intron and UTR alignments. These were then used to generate gCF and sCF values using the neutral phylogenetic model from the “default” dataset as a reference. We observe generally high concordance values between each viviparous taxon and its closely related non-viviparous relatives, with the exception of a few samples within rapidly radiating spiny-finned fish clades known to exhibit higher discordance levels [173, 174, 181].

We ran a total of five PAML models: three that tested for positive selection among branches (i.e., among viviparous species), and two that tested for selection on sites among branches (i.e., the branch-site model).

To test the robustness of these results, we tested for evidence of positive selection on the protein-coding alignments above using BAli-Phy v3.6.0 [140]. We ran BAli-Phy using the branch-site substitution model and the unrooted species tree and viewed the results in Tracer v1.7.2 [182]. Evidence of positive selection was inferred only in the instance in which results were deemed significant by both PAML and BAli-Phy.

To determine whether the acceleration of coding elements in viviparous species could be due to positive selection, we tested for selection on sites in viviparous-accelerated CDS using PAML, as above. For each alignment, we ran both the full and null branch-site models. All alignments were run using the neutral phylogenetic tree with foreground branches set to the viviparous species displaying acceleration for that particular element. We again tested the robustness of these results using the branch-site model in BAli- Phy, as above.

### Supplementary Information

**Additional file 1.** Supplementary Information.

**Additional file 2: Table S1.** Reproductive and genomic information for species included in this study.

**Additional file 3: Figure S1.** Distribution of p-values from the phylogenetically corrected linear mixed model (pglmm) investigating the correlation between viviparity and protein family size. Each dot corresponds to the p-value from a unique protein family.

**Additional file 4: Figure S2.** Time trees. Dated phylogenetic trees of taxa from the "extended" dataset, generated using either maximum (A) or minimum (B) ages of divergence. Species names are displayed as genera. Time is measured in millions of years.

**Additional file 5: Table S2.** Correlation between protein family size and viviparity.

**Additional file 6: Figure S3.** Tree topology for Ubi-N-Sde2. Phylogenetic tree generated using sequences corresponding to Ubi-N-Sde2. Tips are colored according to reproductive mode. Those with an asterisk outline viviparous species showing expansion for Ubi-N-Sde2.

**Additional file 7: Figure S4.** Expansion of the Ubi-N-Sde2 protein family in mammals. (A) The ubiquitin and ubiquitin- like genes which contain Ubi-N-Sde2 fragments for the human, opossum, and platypus. (B) The nucleotide sequence alignment of orthologous *UBC* genes in the human, opossum, and platypus, with gaps displayed in gray and the position of each Ubi-N-Sde2 motif highlighted in blue (in viviparous mammals) and yellow (in oviparous mammals).

**Additional file 8: Table S3.** Alignment statistics for whole-genome alignments generated using different alignment methods.

**Additional file 9: Figure S5.** Concordance factors represented on the neutral phylogenetic model. Node labels correspond to gene concordance factors and site concordance factors generated from intron and UTR (A) and coding sequence alignments (B). Species names are displayed as genera.

## Data Availability

All sequencing data generated in this project is available at NCBI under BioProject PRJNA1066408 [52]. Data and scripts used for processing and analysis are available at Dryad [183].

## Abbreviations

AP3B1: Adaptor related protein complex 3 subunit beta 1
Bdkrb2: Bradykinin B2 receptor
BF1: Bayes factor 1
BF2: Bayes factor 2
b-sheet-shell-Vtg: Beta-sheet shell domain of vitellogenins
CDS: Protein-coding sequence
EVX1: Even-Skipped Homeobox 1
gCF: Gene concordance factor
GO: Gene ontology
pglmm: Phylogenetic generalized linear mixed model
r1: Conservation rate
r2: Accelerated rate
sCF: Site concordance factor
SP5: Sp5 transcription factor
UBA52P1: Ubiquitin A-52 residue ribosomal protein fusion product 1 pseudogene 1
UBA52P5: Ubiquitin A-52 residue ribosomal protein fusion product 1 pseudogene 5
UBBP4: Ubiquitin B pseudogene 4
UBC: Ubiquitin-C
Ubi-N-Sde2: Ubiquitin fold N-terminal domain of silencing defective 2
UTRs: Untranslated regions
ZNF521: Zinc finger protein 521
