# Supplementary material for: Convergent genomic signatures associated with vertebrate viviparity
Source: BMC Biol. 2024 Feb 8;22:34. doi: 10.1186/s12915-024-01837-w (PMC10854053; doi:10.1186/s12915-024-01837-w)
Supplement: Supplementary file 1 — Additional file 1. Supplementary Information. [file 12915_2024_1837_MOESM1_ESM.docx]

**Supplementary Information for**

Convergent genomic signatures associated with vertebrate viviparity

Rhiannon V. Eastment^1^*, Bob B.M. Wong^1^†, Matthew D. McGee^1^‡

^1^School of Biological Sciences, Monash University, Melbourne, 3800, Australia.

*Corresponding author. Email: rhiannon.eastment@monash.edu

† Email: bob.wong@monash.edu

‡ Email: matt.mcgee@monash.edu

# Supplementary Materials and Methods

## Sequencing and assembly of new teleost genomes

Several teleost fishes were obtained from various sources for the sequencing and assembly of new genomes. A *Phanerodon vacca* specimen was obtained from a licensed California scientific collector (Matsu Collections, Inc.). A *Heteroclinus perspicillatus* specimen was obtained from Museums Victoria. A *Xenotoca eiseni* specimen from a long-term captive-bred Australian population was obtained from the aquarium trade (Aquarium Industries, Inc). *Pterois antennata* and *Crossosalaris macrospilus* were obtained from the Australian marine aquarium trade (Cairns Marine, Inc). A freshly caught *Hyporhamphus melanochir* was donated by recreational fishers in Geelong, VIC. Lastly, ethanol- preserved tissue from *Zenarchopterus caudovittatus* was obtained from the Museum and Art Gallery in the Northern Territory of Australia. All live fishes obtained for this study were humanely killed with an overdose of clove oil, with tissue samples preserved in chilled 99% propylene glycol (Monash University AEC 17725).

## Whole genome alignments

We generated a multiple-genome alignment comprising 27 vertebrate genomes using LAST v1256 [20]. LAST is unlike traditional alignment tools, such as BLAST and LASTZ, which use a ‘seed-and-extend’ approach to find short, initial matches, or ‘seeds’ [21-23]. Instead, LAST uses adaptive seeds, which are identified on account of their rareness, as opposed to their length [20,24]. In doing so, LAST identifies only the strongest matches and thus outperforms other aligners in both runtime and sensitivity [20].

Of the 27 genomes used in this alignment—which we now refer to as the ‘default’ alignment—20 were obtained from NCBI, and seven were sequenced and assembled for the purposes of this project. Existing genomes were chosen based on their quality, availability, and phylogenetic relationship with one another, in that each viviparous species was paired up with a closely related oviparous species [19,25,26]. All additional oviparous taxa were then chosen on account of their more distant evolutionary relationship to each pair.

We generated an additional, ‘extended’ alignment to increase taxonomic diversity and provide more comprehensive sampling for an analysis in which we compared the size of protein families among viviparous and oviparous species. This ‘extended’ dataset comprised the 27 genomes from the ‘default’ dataset above, and an additional 24 genomes obtained from NCBI.

***Establishing alignment parameters***

To test the robustness and efficiency of our alignment methodology, we carried out a series of additional alignments, each time altering either the seeding scheme used to prepare the reference genome, or the reference genome itself (see Additional file 8: Table S3). We trialed three different seeding schemes from LAST: MAM8, NEAR, and YASS. MAM8 is a highly sensitive scheme that finds weak similarities between sequences [24]. NEAR identifies highly similar sequences with many gaps, while YASS identifies long sequences with weak similarities. We additionally aligned all 27 genomes from the ‘default’ dataset to two alternative reference genomes, both of which were obtained from NCBI (*Amia calva:* GCA_017591415.1 and *Petromyzon marinus:* GCF_010993605.1) [27].

MafFilter v1.3.1 was used to generate alignment statistics for each of the additional alignments [28], which are presented in Additional file 8: Table S3. Ultimately, the *H. sapiens* genome was assigned as the reference due to its high quality, indicated by its level of annotation, completeness, and correctness, and was prepared using the MAM8 seeding scheme.

## Extraction of coding and noncoding regions

MafFilter v1.3.1 [28] was used to obtain 892 CDS alignments from the ‘default’ dataset and 219 from the ‘extended’ dataset. 1,673 noncoding sequence alignments were extracted from the ‘default’ dataset, and 517 from the ‘extended’ dataset. After accounting for duplication and overlap, we obtained a total of 858 coding alignments and 1,598 noncoding alignments from the ‘default’ dataset, and 219 coding alignments and 491 noncoding alignments from the ‘extended’ dataset. The resulting alignments were then realigned to account for errors introduced during the initial alignment. Although ‘maf-join’ takes the coordinates of the reference genome into account when joining pairwise alignments, it arbitrarily joins alignment columns when there are gaps in the reference and could therefore cause errors in the final alignments [20].

## Species tree inference

The GTR model allows for different nucleotide frequencies and substitution rates and is thus more parameter-rich, making it better suited to large data sets, such as our own [29,30]. We generated additional tree topologies for the ‘default’ dataset using either the entire alignment, or protein-coding sequence alignments, as above. Both the whole-genome alignment and coding sequence alignment gave the incorrect placement for the lobe-finned fishes [19]. We suspect that the use of coding sequences gave the incorrect placement for the lobe-finned fishes, as opposed to the noncoding sequences, for several reasons. Firstly, there were twice as many intron and UTR sequence alignments (n=1,598) than there were coding alignments (n=858), providing a greater sample size in which to infer phylogenetic relationships. Lastly, noncoding data has been suggested to be more useful in inferring phylogenetic relationships than coding sequences, based on recent studies [31]. Ultimately, we chose to use molecular data (that is, introns and UTRs) that were in agreement with paleontological records.

## Mapping the evolution of Ubi-N-Sde2

We traced the location of each Ubi-N-Sde2 sequence in the human (*H. sapiens*), opossum (*G. agilis*) and platypus (*O. anatinus*) genomes using the UCSC Genome Browser [32]. Doing so revealed Ubi-N-Sde2 motifs in Sde2, as was expected, but also in many ubiquitin and ubiquitin-like genes. We therefore investigated insertion and/or transposition of Ubi-N-Sde2 motifs as a potential reason for their presence in ubiquitin-like genes in viviparous mammals.

For each gene containing sequence fragments of Ubi-N-Sde2, we identified orthologous genes in the remaining mammals using a combination of genome annotation data and sequence alignments. We first used the UCSC Genome Browser to search for the relevant gene in each of the 3 mammalian genomes. In instances where multiple transcripts were found, we obtained coordinates corresponding to the longest one. In instances where genes could not be found using the Genome Browser, minimap2 was used to find orthologous genes by mapping the gene of interest to the target genome [33]. Minimap2 hits with a mapping quality <30 and length <200bp were excluded. Where multiple hits resulted from mapping, the sequence with the best mapping quality score and largest length of alignment was considered the most likely ortholog. Where orthology could not be confirmed using existing annotation information and minimap2, we inferred orthology using the ‘maf-cut’ program in LAST [20] to identify sequence alignments of the gene of interest from the multiple- genome alignments generated above.

***PhyloAcc analysis***

We ran PhyloAcc 3 times each for the coding and noncoding datasets with default parameters, each time altering the target species. The targets were set to viviparous species for run 1, oviparous species for run 2, and placental species for run 3 - the identities of which are outlined in Additional file 2: Table S1.

***Gene ontology enrichment***

GOrilla was run twice for the accelerated coding and noncoding regions of interest using the *H. sapiens* database. The first run used the complete list of target and background genes, which contained some duplicates due to the presence of antisense genes. We then ran GOrilla again using a refined list of genes which excluded antisense genes. There were no differences in the enriched terms between runs. 3 GO terms were enriched amongst accelerated noncoding regions, while no enrichment was found amongst accelerated coding regions.

## Positive selection on protein-coding regions

We looked for evidence of positive selection on coding alignments using the codeml program in PAML v4.9 [34]. A above, we first ran model M0, which fits a single dN/dS to each branch. We then ran the free-ratio branch model, which fits a unique dN/dS to each branch. Finally, we ran model M2, which fits a separate dN/dS to foreground branches (i.e., viviparous species) and background branches (i.e., oviparous species). We then constructed likelihood ratio tests comparing models M0 and M2, and models M1 and M2. Chi-square tests were performed for each to detect positive selection (indicated by P < 0.05).

To detect positive selection among all viviparous species at sites across each alignment, we ran the full branch-site model (in which omega is estimated) and the null branch-site model (in which omega is fixed and is equal to 1).
