# Supplementary material for: Convergent genomic signatures associated with vertebrate viviparity
Source: BMC Biol. 2024 Feb 8;22:34. doi: 10.1186/s12915-024-01837-w (PMC10854053; doi:10.1186/s12915-024-01837-w)
Supplement: Supplementary file 3 — Additional file 3: Figure S1. Distribution of p-values from the phylogenetically corrected linear mixed model (pglmm) investigating the correlation between viviparity and protein family size. Each dot corresponds to the p-value from a unique protein family. [file 12915_2024_1837_MOESM3_ESM.docx]

**
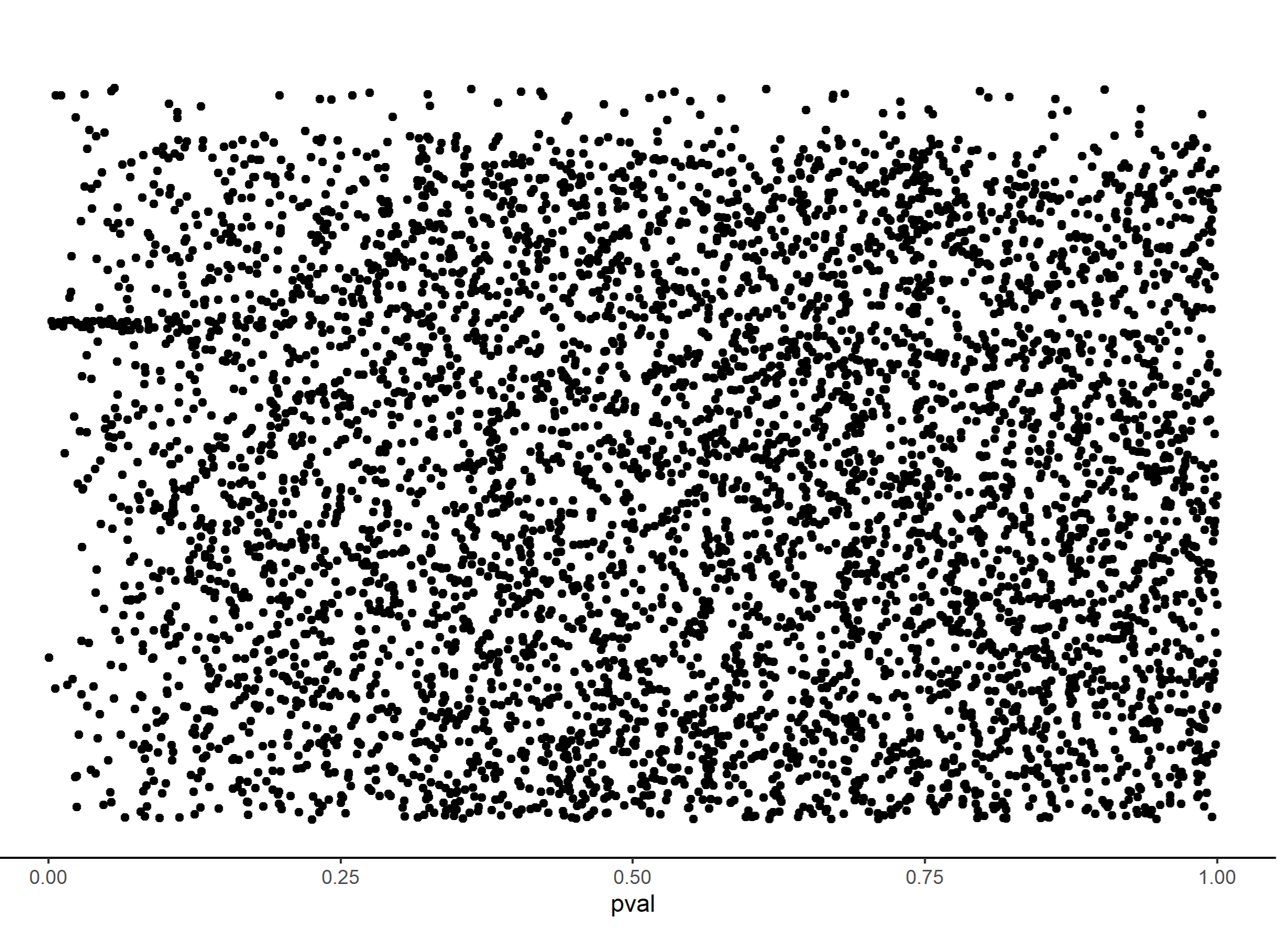
**

**Figure S1. Distribution of p-values from the phylogenetically corrected linear mixed model (pglmm) investigating the correlation between viviparity and protein family size.** Each dot corresponds to the p-value from a unique protein family.
