# Supplementary material for: Convergent genomic signatures associated with vertebrate viviparity
Source: BMC Biol. 2024 Feb 8;22:34. doi: 10.1186/s12915-024-01837-w (PMC10854053; doi:10.1186/s12915-024-01837-w)
Supplement: Supplementary file 4 — Additional file 4: Figure S2. Time trees. Dated phylogenetic trees of taxa from the "extended" dataset, generated using either maximum (A) or minimum (B) ages of divergence. Species names are displayed as genera. Time is measured in millions of years. [file 12915_2024_1837_MOESM4_ESM.docx]

**B**

**A**

**
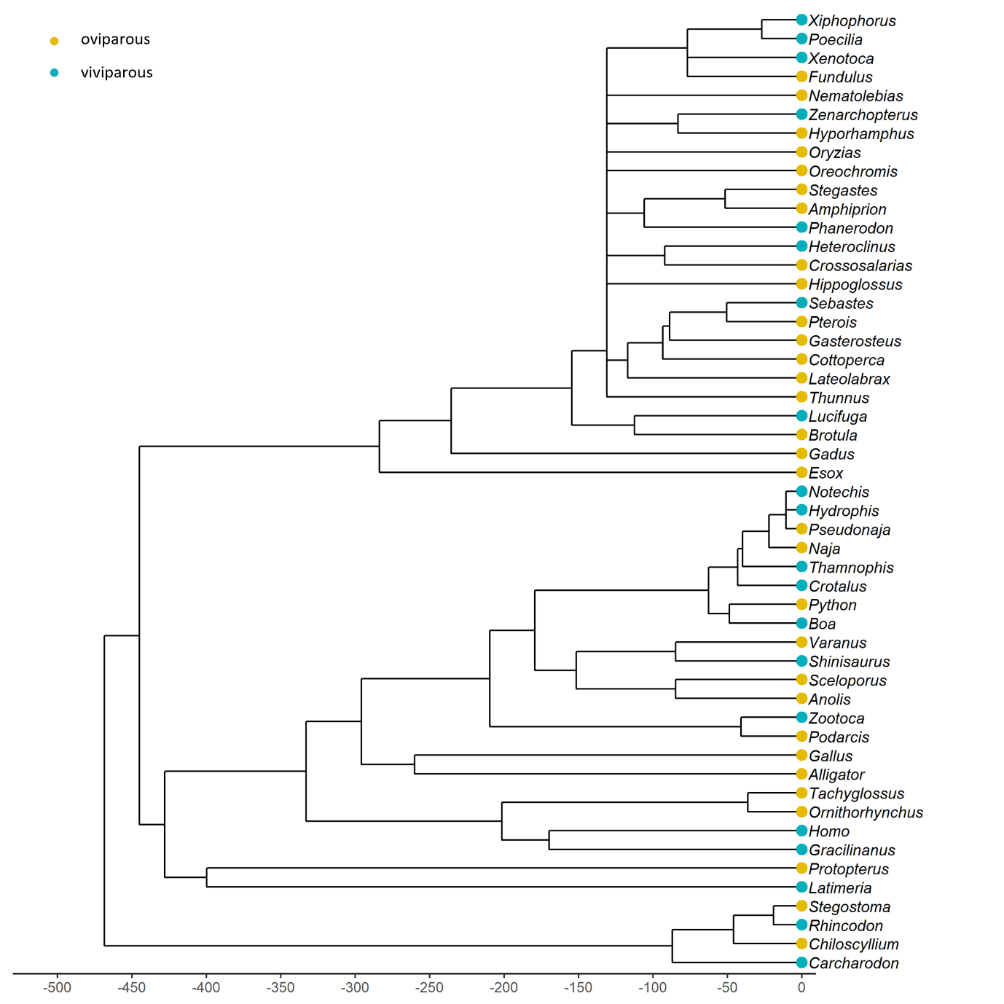

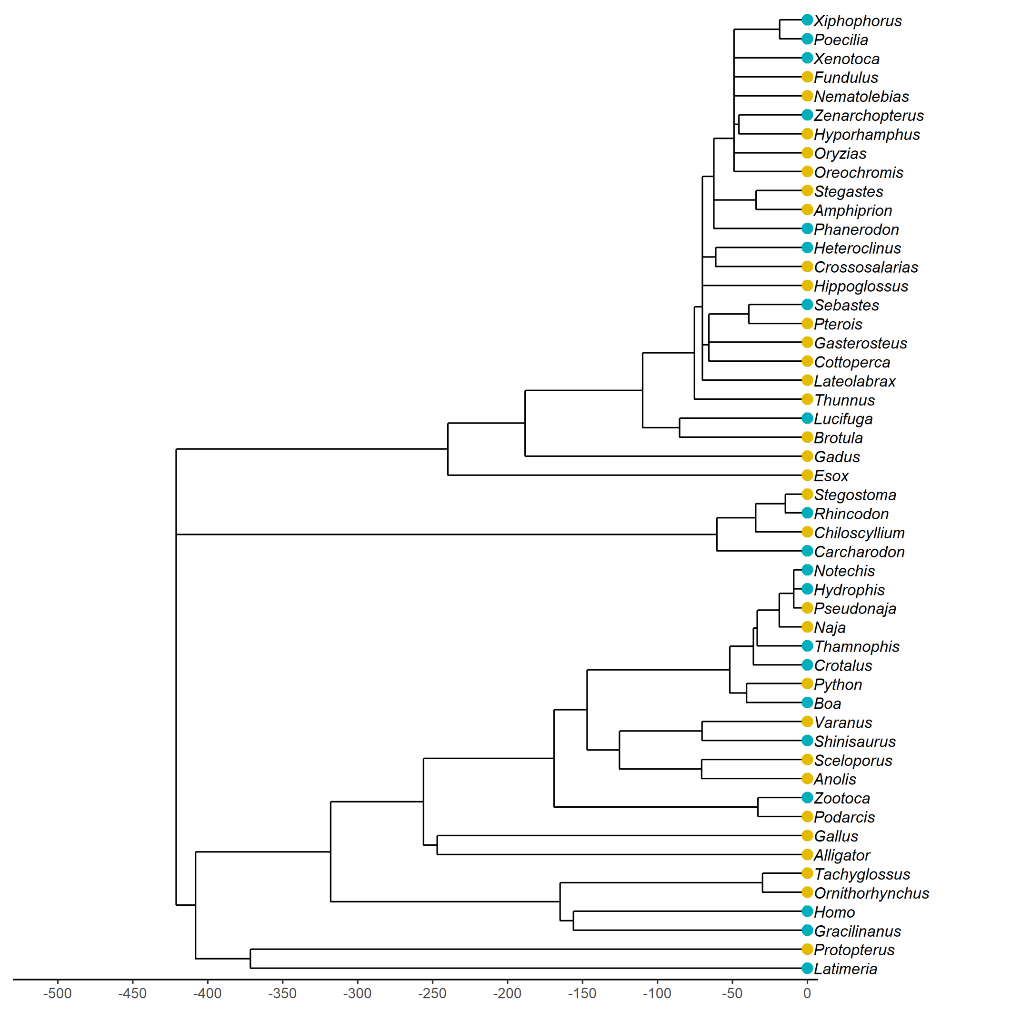
**

**Figure S2. Time trees.** Dated phylogenetic trees of taxa from the ‘extended’ dataset, generated using either maximum (A) or minimum (B) ages of divergence. Species names are displayed as genera. Time is measured in millions of years.
