# Supplementary material for: Convergent genomic signatures associated with vertebrate viviparity
Source: BMC Biol. 2024 Feb 8;22:34. doi: 10.1186/s12915-024-01837-w (PMC10854053; doi:10.1186/s12915-024-01837-w)
Supplement: Supplementary file 6 — Additional file 6: Figure S3. Tree topology for Ubi-N-Sde2. Phylogenetic tree generated using sequences corresponding to Ubi-N-Sde2. Tips are colored according to reproductive mode. Those with an asterisk outline viviparous species showing expansion for Ubi-N-Sde2. [file 12915_2024_1837_MOESM6_ESM.docx]

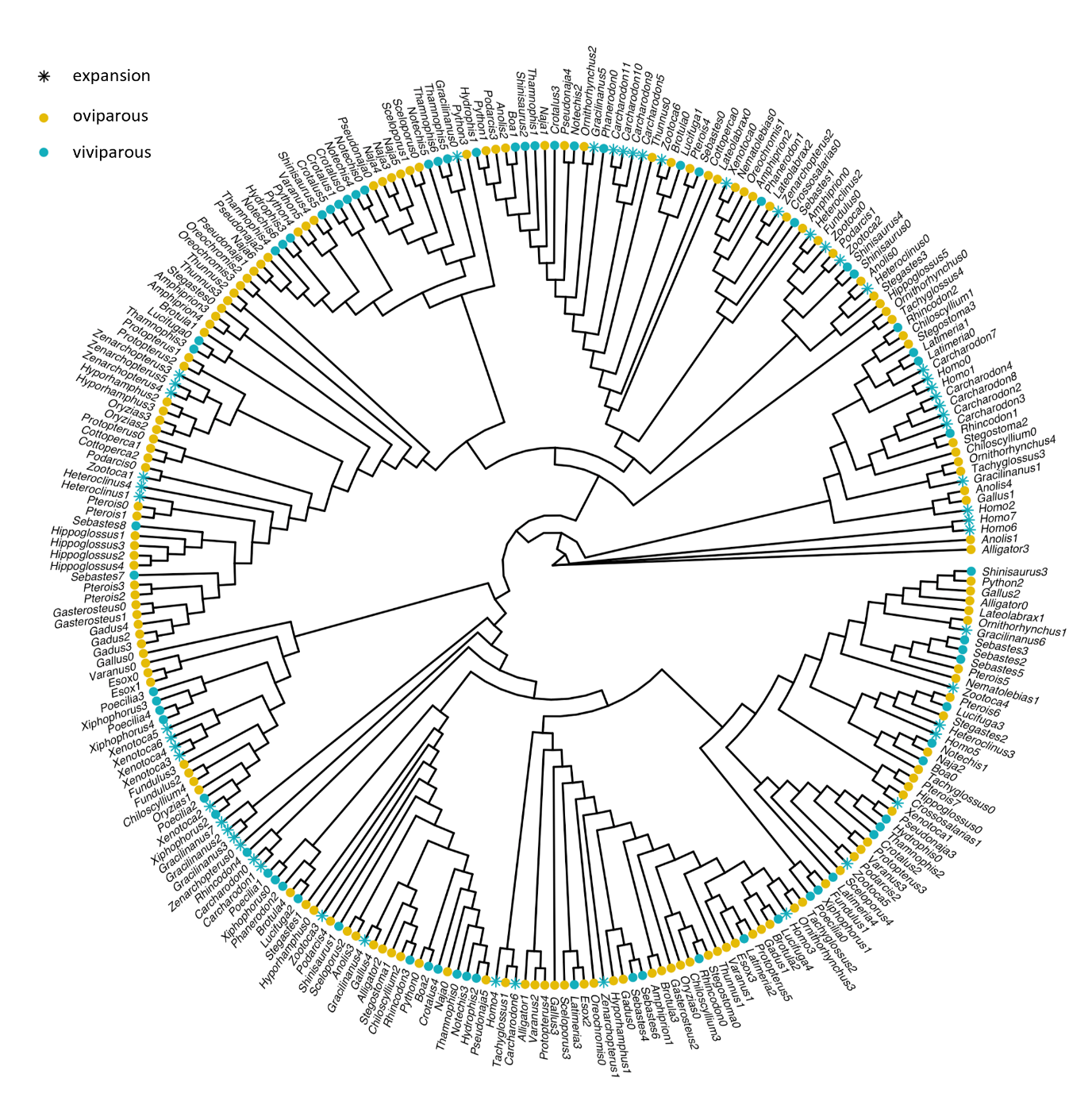


**Figure S3. Tree topology for Ubi-N-Sde2.** Phylogenetic tree generated using sequences corresponding to Ubi-N-Sde2. Tips are colored according to reproductive mode. Those with an asterisk outline viviparous species showing expansion for Ubi-N-Sde2.
