# Supplementary material for: Convergent genomic signatures associated with vertebrate viviparity
Source: BMC Biol. 2024 Feb 8;22:34. doi: 10.1186/s12915-024-01837-w (PMC10854053; doi:10.1186/s12915-024-01837-w)
Supplement: Supplementary file 7 — Additional file 7: Figure S4. Expansion of the Ubi-N-Sde2 protein family in mammals. (A) The ubiquitin and ubiquitin- like genes which contain Ubi-N-Sde2 fragments for the human, opossum, and platypus. (B) The nucleotide sequence alignment of orthologous UBC genes in the human, opossum, and platypus, with gaps displayed in gray and the position of each Ubi-N-Sde2 motif highlighted in blue (in viviparous mammals) and yellow (in oviparous mammals). [file 12915_2024_1837_MOESM7_ESM.docx]

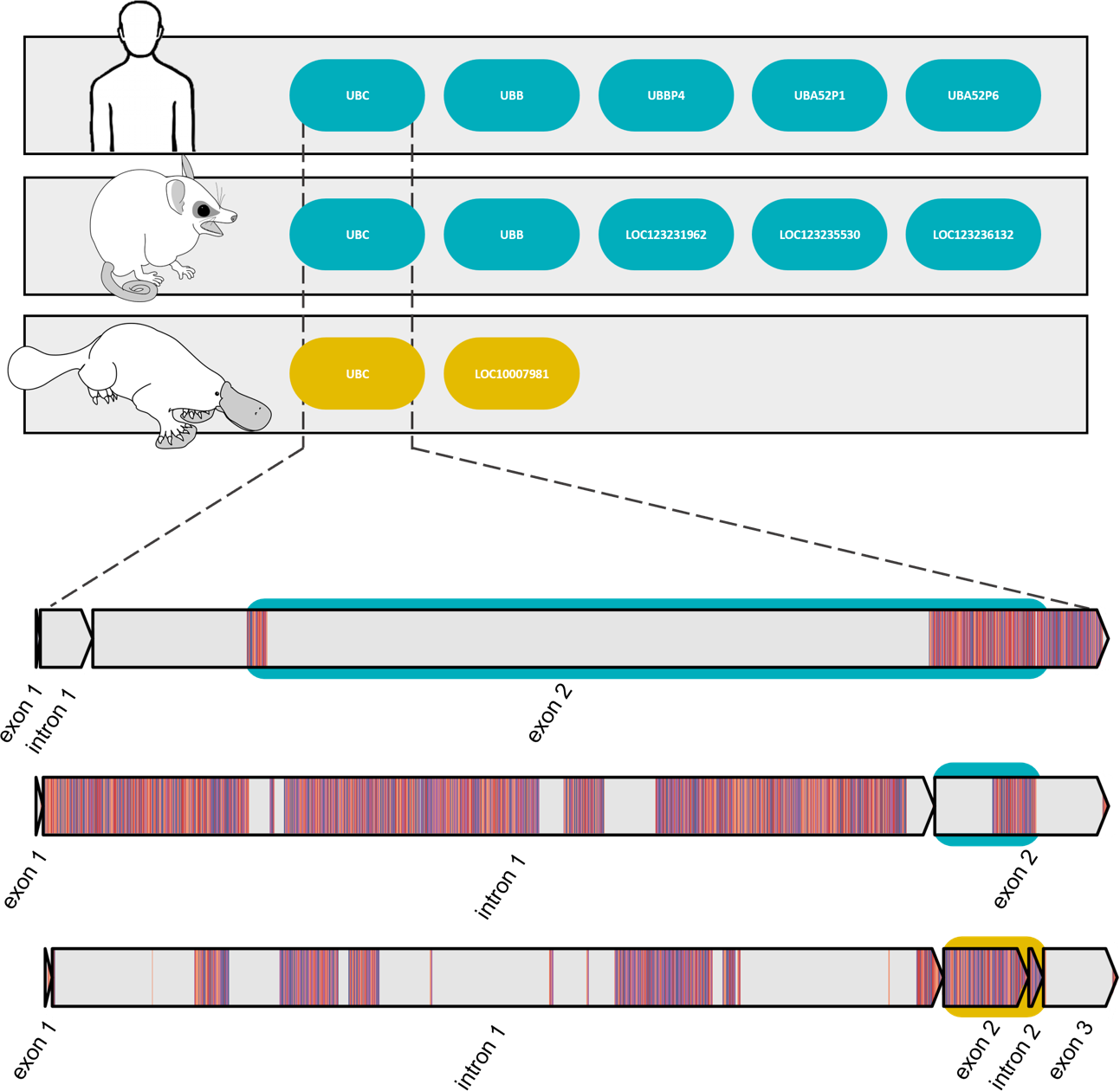


**A**

**B**

**Figure S4. Expansion of the Ubi-N-Sde2 protein family in mammals.** (A) The ubiquitin and ubiquitin- like genes which contain Ubi-N-Sde2 fragments for the human, opossum, and platypus. (B) The nucleotide sequence alignment of orthologous UBC genes in the human, opossum, and platypus, with gaps displayed in gray and the position of each Ubi-N-Sde2 motif highlighted in blue (in viviparous mammals) and yellow (in oviparous mammals).
