# Supplementary material for: Convergent genomic signatures associated with vertebrate viviparity
Source: BMC Biol. 2024 Feb 8;22:34. doi: 10.1186/s12915-024-01837-w (PMC10854053; doi:10.1186/s12915-024-01837-w)

**Figure S5. Concordance factors represented on the neutral phylogenetic model.** Node labels correspond to gene concordance factors and site concordance factors generated from intron and UTR (A) and coding sequence alignments (B). Species names are displayed as genera.
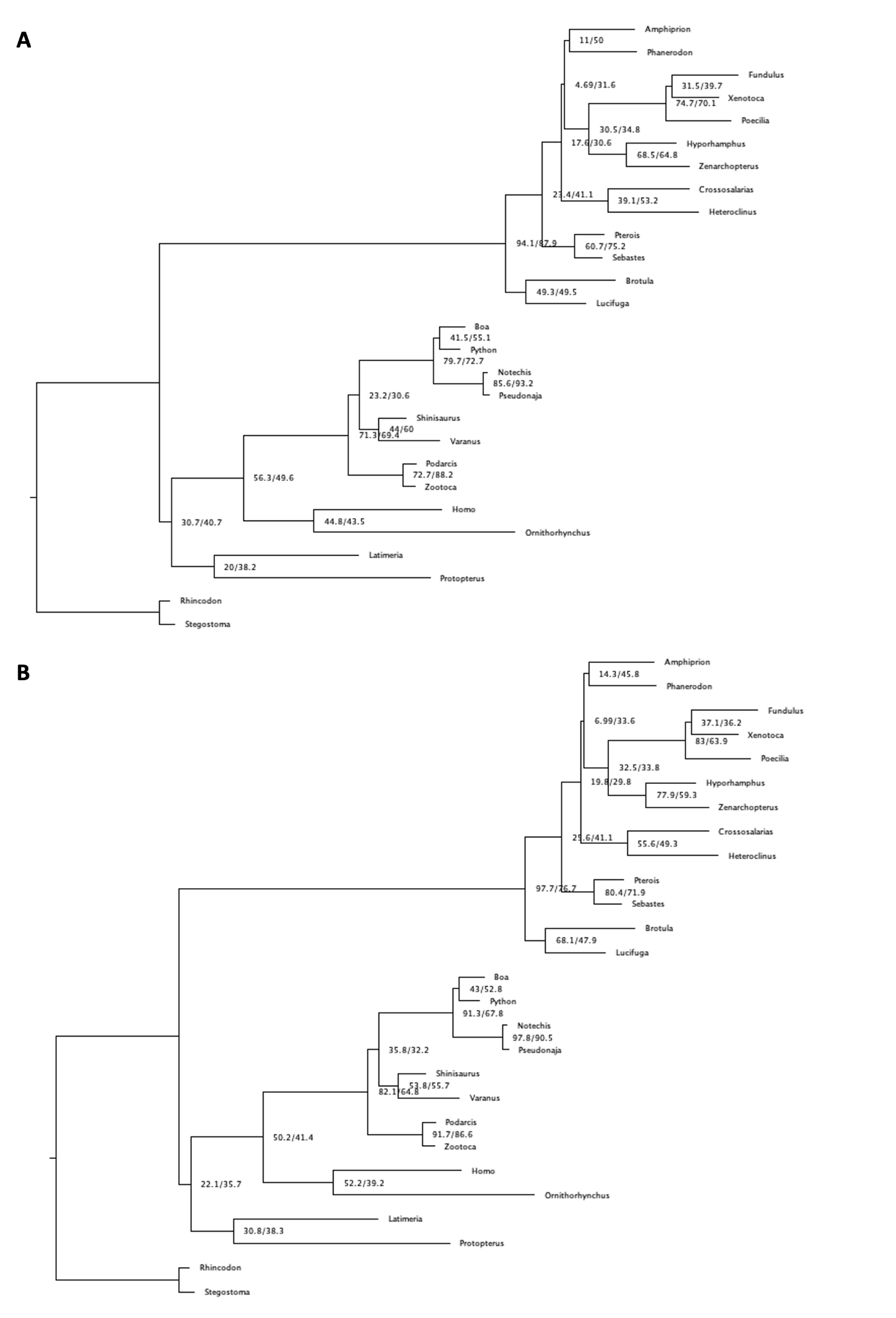

Supplement: Supplementary file 9 — Additional file 9: Figure S5. Concordance factors represented on the neutral phylogenetic model. Node labels correspond to gene concordance factors and site concordance factors generated from intron and UTR (A) and coding sequence alignments (B). Species names are displayed as genera. [file 12915_2024_1837_MOESM9_ESM.docx]
